# Continuation of self-injected versus provider-administered contraception in Senegal: a nonrandomized, prospective cohort study^☆^^☆☆^^★^

**DOI:** 10.1016/j.contraception.2018.11.001

**Published:** 2019-02

**Authors:** Jane Cover, Maymouna Ba, Jennifer Kidwell Drake, Marėme Dia NDiaye

**Affiliations:** a PATH, PO Box 900922, Seattle, WA 98109, USA; b PATH, BP 15115, Dakar-Fann, Dakar, Senegal; c Ministère de la Santé et de l'Action Sociale, Dakar, Senegal

**Keywords:** Self-injection, Self-administration, Injectable contraception, Depot-medroxyprogesterone acetate, Subcutaneous DMPA, DMPA-SC

## Abstract

**Objectives:**

The primary objective of this study was to compare the 12-month continuation rate for women who self-injected subcutaneous depot-medroxyprogesterone acetate (DMPA-SC) with that for women receiving intramuscular depot-medroxyprogesterone acetate (DMPA-IM) from a provider. This research contributes to the broader goal of identifying solutions to support women to use contraception for their full desired duration.

**Study design:**

Participants were clients from 13 clinics in the Dakar and Thiés regions of Senegal who had decided to use injectable contraception prior to enrollment. They chose self-injection of DMPA-SC or provider administration of DMPA-IM. Self-injectors were trained and given three units of DMPA-SC. The provider-injected group received DMPA-IM and returned to the clinics for future injections. We interviewed participants at baseline and after the second, third and fourth injections (the equivalent of 12 months of contraceptive coverage). We employed Kaplan–Meier methods to estimate continuation probabilities, with a log-rank test to compare differences between groups. A multivariate Cox regression identified factors correlated with discontinuation.

**Results:**

The 12-month continuation rate for 650 women self-injecting DMPA-SC was 80.2%, while that for 649 women receiving DMPA-IM from a provider was 70.4% (p<.01). The difference in continuation between self-injectors and those receiving DMPA from a provider remained significant in a multivariate Cox regression model. The primary reason for discontinuation in both groups (44.7% self-injected; 44.5% provider-injected) was forgetting to reinject or reinjecting late. Fewer women reported side effects in the self-injection group than in the provider-administered group.

**Conclusions:**

The higher 12-month continuation rate for women self-injecting DMPA-SC relative to provider-administered DMPA-IM suggests that self-injection may help prevent pregnancy more consistently and continuously.

**Implications:**

Discontinuation of injectable contraception among women wishing to avoid pregnancy may increase unmet need in francophone West Africa. This study showed higher 12-month continuation rates for women who self-injected DMPA-SC, suggesting that this delivery method may improve injectable continuation.

## 1 Introduction

Francophone West Africa is a region with persistently high fertility and total fertility rates ranging from 4.5 (Togo) to 7.3 (Niger) children per woman [1]. While Senegal saw a substantial increase in modern contraceptive use from 12% to 23% from 2011 to 2016, the total fertility rate remains high at 4.7 children per woman [2]. The injectable — most commonly, depot medroxyprogesterone acetate (DMPA) — is the most popular method, representing over one third of the method mix in 2016 [2]. Though injectable contraception is effective, nearly half of women in Senegal (42%) discontinue the method within 12 months of initiation [2]; this pattern of short average duration of injectable use is consistent with findings from other countries [3].

A newly available product, subcutaneous DMPA (DMPA-SC) in the Uniject™ injection system, has the potential to improve continuity of use. DMPA-SC contains a lower dose of DMPA and is packaged in an easy-to-use, all-in-one injection system, making it possible for women to self-inject. Self-injection not only reduces the burden of quarterly return visits to clinics but also enhances women decision making and autonomy with respect to contraceptive use. This product (brand name Sayana® Press, manufactured by Pfizer) received regulatory approval in Senegal in 2014. Sayana Press was approved for self-injection by the United Kingdom Medicines and Health Regulatory Agency in 2015, and self-injection labeling is currently being reviewed by the regulatory authority in Senegal. A self-injection study in Senegal in 2016 found that 87% of women were able to self-inject competently 3 months posttraining [4]. More than 9 in 10 women (93%) reported that they would continue with self-injection if it were available in the future [4]. Based on these findings, the Senegal Ministry of Health is moving forward with provider training to scale up self-injection as a delivery modality.

While women in diverse settings view self-injection favorably because of the savings in time and travel expenses, the extent to which self-injection improves outcomes for family planning programs is less clear [4], [5], [6], [7], [8]. The first randomized control trial of self-injection in the United States, in which 12-month continuation was an outcome measure, found somewhat higher continuation among women self-injecting compared with women receiving DMPA-SC from a health care provider; however, the difference did not reach statistical significance [9]. A subsequent review of the literature concluded that there was no evidence of improved continuation for women who self-inject [10]. However, recent studies from Malawi, Uganda and the United States suggest that self-injection may improve injectable continuation [11], [12], [13]. While these studies are promising, it remains to be seen whether self-injection improves continuation in the high-fertility setting of francophone West Africa.

The primary objective of this study was to compare the rate of continuation for women who self-inject DMPA-SC with that of women receiving DMPA-IM from a provider. The secondary objectives were to identify differences in the characteristics of participants who opt for self-injection compared with women who choose provider-administered DMPA-IM and to identify factors that contribute to discontinuation of injectable contraception more broadly. Meeting these objectives will contribute to the broader goal of identifying solutions to support women to use contraception for their full desired duration.

## 2 Materials and methods

### 2.1 Study design, sites and participants

This was a nonrandomized, prospective cohort study implemented by licensed nurses trained in DMPA-SC and DMPA-IM administration, research ethics, interviewing techniques and how to counsel women for self-injection. All participants were family-planning clients at 1 of 13 clinics in the Dakar and Thiés regions of Senegal. These sites were purposively chosen to capture variations in urban/rural geography and included four rural sites, four periurban sites and five urban sites. We collected data from October 2016 to December 2017.

Participants were women 18 to 49 years of age who had decided to use injectable contraception prior to study enrollment, were eligible for DMPA use per World Health Organization (WHO) medical eligibility guidelines and wanted to avoid pregnancy for at least 12 months. All participants provided written informed consent and understood French or Wolof.

### 2.2 Study procedures

All women who selected injectable contraception and were interested in participating in the study were given the option to try self-injection of DMPA-SC or to receive DMPA-IM injections from a provider. Study staff assessed eligibility of potential participants and enrolled women in the groups of their choice. Urine pregnancy tests confirmed that participants were not pregnant at the time of enrollment.

Study nurses trained women in the self-injection group individually and evaluated their self-injection competency using an observation checklist. For clients to be considered competent at self-injection, they were required to correctly complete five critical injection steps on the observation checklist (see supplemental material). Study nurses gave three DMPA-SC units, an instruction booklet and a reinjection calendar to those judged to be competent. Those not competent were asked to return at the time of their second injection for refresher training, at which time their competency was reassessed and, if competent, they were given self-injection supplies. The provider-injected DMPA-IM group received injections at the clinic and was given appointment cards to return for future injections.

To minimize loss to follow-up, all women in the study provided detailed contact information upon enrollment, including phone numbers and addresses (with visual maps constructed by the study staff) to facilitate follow-up via phone calls and home visits. We interviewed all participants after the second, third and fourth injections (the equivalent of 12 months of contraceptive coverage) to assess continuation. Women in the provider-injected group were interviewed at each return visit to the health facility after receiving the injection from a provider. If women in the provider-injection group did not return for reinjection within 29 days following their scheduled reinjection date (close of the WHO-approved reinjection window for DMPA [14]), study nurses followed up for an interview at home or other locale of their choosing. Study nurses interviewed those in the self-injection group 29 days after their scheduled reinjection dates. Women were queried as to whether they were continuing and the date of their most recent injection, as well as the scheduled date of their next injection; their experience regarding side effects and injection-site reactions; their experience of self-injection or of their return clinic visit (if receiving injections from a provider); their satisfaction with the method and their intention to continue. Woman who discontinued were asked their reasons for discontinuation, their fertility intentions and their current method use or future contraceptive plans.

### 2.3 Sample size

The required sample size was 654 in each group. This assumes a power of 90%, a significance level of .05 and a 10-percentage-point difference in continuation rates between provider-injected DMPA-IM users and those self-injecting DMPA-SC. The sample size assumed a loss to follow-up rate of 20%.

### 2.4 Data collection and analysis

We collected data via private, in-person structured interviews conducted in either French or Wolof according to the preference of the client. We analyzed data with Stata, version 14.

The primary endpoints of the study were 12-month continuation rates, defined as receiving four consecutive on-time injections. We considered as discontinued women who self-injected or received any injection more than 120 days after the previous injection (to accommodate the WHO 4-week reinjection window [14]). Women who were judged not competent with the injection technique at the time of the second injection and women lost to follow-up were discontinued from the study. Consistent with an intent-to-treat approach, we classified as continuing in the provider-injected group participants who switched from DMPA-IM to DMPA-SC (since both were given by a provider).

We estimated cumulative contraceptive continuation over the 12-month study period using Kaplan–Meier methods, with continuation censored for those who received the 9-month injection, which provided 12 months of contraceptive coverage. We used the log-rank test to estimate equality of the survival function between injection groups. Differences in background characteristics and side effects between the two groups were evaluated via *χ*^2^ tests or *t* tests, with a significance threshold of .05 for a two-sided test. Cox proportional-hazard models were used to identify significant predictors of discontinuation in a multivariate model.

### 2.5 Confidentiality and ethical approvals

The Research Ethics Committee at PATH and the Comité National d'Ethique pour la Recherche en Santé in Senegal granted approval for this research study.

## 3 Results

In all, 1299 women enrolled in the study: 650 in the DMPA-SC self-injection group and 649 in the provider-administered DMPA-IM group. Baseline data for one individual in the self-injection group were lost, but her data were retained in the analysis for the continuation rate. A total of 16 participants (6 in the self-injection group and 10 in the provider-injected group) were lost to follow-up and considered to have discontinued 90 days after their last recorded injection. Consistent with an intent-to-treat approach, 82 women in the provider-injected DMPA-IM group who switched to DMPA-SC administered by a provider were retained in the provider-injected DMPA-IM group as continuers. Three women were judged not competent at self-injection after two training sessions and were discontinued by the study staff at the time of their second injection.

### 3.1 Participant background

The profiles of self-injectors and those receiving injections from providers differed somewhat at baseline (Table 1). Self-injectors tended to have higher socioeconomic status, with more years of education and higher household assets scores, and had experience with DMPA-SC administered by a provider. Participants who received injections from providers had more children on average, were more likely to report paying to travel to the clinic and reported more needle anxiety.

**Table 1:** Sociodemographic characteristics of participants who self-injected DMPA-SC (*n*=649) or received DMPA-IM injected by a provider (n=649) in Senegal

	Self-injected DMPA-SC (*n*=649^a^)		Provider-injected DMPA-IM (*n*=649)		p value
	% or mean	*n*	% or mean	*n*	
Mean age (SD)	28.8 (6.2)	649	29.4 (6.3)	649	.11
Married or cohabiting	97.7	634	98.2	637	.56
Mean parity (SD)	2.8 (1.8)	649	3.2 (1.9)	649	.00
Education level				331	.00
None	37.1	241	51.0	177	
Primary	31.6	205	27.3	127	
Secondary	25.3	164	19.6	14	
University	6.0	39	2.2		
Working outside the home	41.5	269	40.7	264	.78
Collects paycheck	10.6	69	10.5	68	.93
Mean number of household assets (SD)	9.9 (3.3)	649	9.5 (3.1)	649	.02
Mean travel time RT to facility, min (SD)	54.0 (47.5)	649	57.2 (45.6)	649	.21
Paid to travel to facility	35.0	227	43.8	284	.00
First-time contraceptive user	10.8	70	11.6	75	.66
Current or past injectable user	87.1	565	85.7	556	.47
Current or past DMPA-SC user	39.5	256	8.6	56	.00
Injection anxiety					.00
Low	70.6	458	50.7	329	
Moderate	24.7	160	29.9	194	
High	4.8	31	19.4	126	
Mean number of methods ever used (SD)	1.4 (0.8)	649	1.3 (0.7)	649	.06
Husband supports use of family planning	87.4	567	85.1	552	.23
Family planning decisions made jointly	59.9	389	55.5	360	.10

SD, standard deviation; RT, roundtrip.

a Baseline data for one self-injecting participant were missing.

### 3.2 Self-injection experience

Among self-injectors, 97% were deemed competent when self-injecting for the first time (data not shown). Self-injectors reported increased facility in giving injections over time, though some nonetheless sought assistance (Table 2). Those who reported difficulty with self-injection identified needle insertion, activation of the device or, more generally, knowledge of the steps as challenging (data not shown). Most used the booklet for guidance, and all but two who did not use the booklet reported not needing it. Fewer women used the calendar, and a sizeable share of those who did not (30%; data not shown) reported difficulty understanding it. While nearly four in five self-injectors could accurately identify their next injection date, fewer women in the provider-injected group could do so (between 67% and 59%, depending on the injection; data not shown.)

**Table 2:** Women's experiences with self-injection in Senegal, including perceived difficulty with self-injection, and practices to aid self-injection

	Injection 2 (*n*=619)	Injection 3 (*n*=589)	Injection 4 (*n*=565)
Reported challenges with device storage prior to injection	6/576^a^ (1.0)	7 (1.2)	10 (1.8)
Reported injection very easy to administer	547 (88.4)	550 (93.4)	548 (97.0)
Sought injection help from providers, family or friends	65 (10.5)	37 (6.3)	21 (3.7)
Used the booklet during self-injection	594 (96.0)	539 (91.5)	480 (85.0)
Used the calendar to schedule next injection	450 (72.7)	429 (72.8)	341 (60.4)
Correctly identified next injection date	479 (77.4)	457 (77.6)	434 (76.8)
Kept used needle in container prior to disposal	417/576^a^ (72.4)	376 (63.8)	311 (55.0)

a Those who returned to the clinic for additional training when due for the second injection were not asked about at-home storage of the needle or disposal postinjection since the unit was not stored at home and was disposed of immediately at the clinic into a safety box.

### 3.3 Continuation

**Fig. 1:**
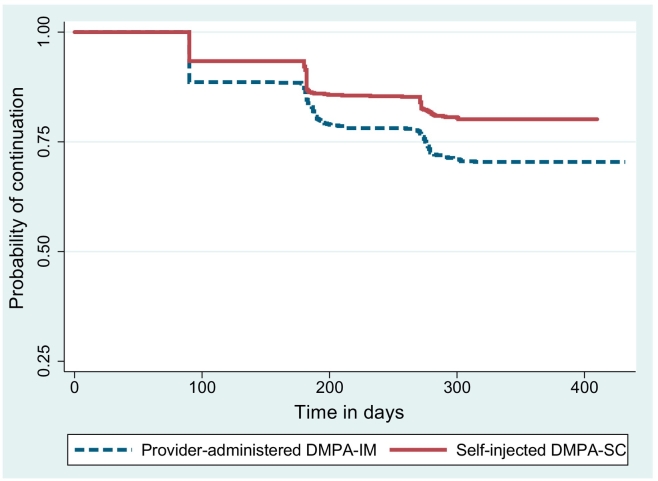
Kaplan–Meier cumulative probability of continuation of contraception. Log-rank test for the equality of survival function, p value <.000.

The 12-month continuation rate (through four injections) for the self-injectors was 80.2% compared with 70.4% for those receiving injections from providers (significant at the p<.001 level). Fig. 1 shows that the probability of continuation was higher among self-injectors at each reinjection time point (~90 days; ~180 days; ~270 days). A sensitivity analysis found that, if we exclude from analysis women who switched type of injectable or mode of administration, the continuation rates were 81.3% for self-injectors and 69.1% for those in the provider-administered group (data not shown).

### 3.4 Determinants of discontinuation

We conducted a Cox proportional-hazard regression analysis with clustering by study site to show the factors associated with discontinuation. As shown in Table 3, factors that increased the hazard for discontinuing were paying for travel to the clinic and experiencing side effects, while factors that decreased this hazard included being a self-injection client, having more children, having a primary or secondary education (relative to no education) and having more household assets. Thus, being a self-injecting client decreased the hazard of discontinuing, relative to provider-injected clients, controlling for age, parity, education, assets, rural location, payment for travel and experiencing side effects.

**Table 3:** Risk of discontinuation of injectable contraception over 12 months in Senegal (*n*=1298)

Variable	Adjusted hazard ratio (95% CI)	p value
Self-injection client	0.72 (0.56–0.93)	.00
Age	1.17 (1.00–1.38)	.06
Age (squared)	1.00 (0.99–1.00)	.07
Parity	0.87 (0.80–0.94)	.00
Education (reference: no education)		
Primary	0.70 (0.53–0.92)	.01
Secondary and higher	0.67 (0.47–0.95)	.03
Total household assets	0.96 (0.92–0.99)	.01
Clinic in a rural location	1.14 (0.74–1.74)	.56
Pay for travel to clinic	1.30 (1.04–1.62)	.02
Experienced side effects	1.69 (1.09–2.63)	.02

Cox proportional-hazard ratios with clustering by study site, predicting risk of contraceptive discontinuation over 12 months.

CI, confidence interval.

### 3.5 Reasons for discontinuation

Women could identify any number of reasons for discontinuing, and the most prominent reason in both groups was forgetting to have the injection or reinjecting late, i.e., after the 4-week reinjection window had closed (Table 4). Discontinuing the injectable to have a baby was the second most common reason in the provider-injected group and the third most common reason in the self-injection group. A small percentage of women in the self-injection group (5.7%) reported that they discontinued due to challenges with self-injection.

**Table 4:** Women's self-reported reasons^a^ for discontinuing self-injected DMPA-SC (*n*=123) or provider-injected DMP-IM (*n*=182) in Senegal

	Self-injected DMPA-SC (*n*=123)		Provider-injected DMPA-IM (*n*=182)	
	%	*n*	%	*n*
Forgot/late for injection	44.7	55	44.5	81
Husband disapproval	23.8	29	9.0	16
To have a baby	21.1	26	18.1	33
Side effects	6.5	8	13.7	25
Challenges with self-injection^b^	5.7	7	--	--
No sexual relations	4.9	6	9.3	17
Access challenges/stockouts^c^	3.3	4	3.9	7
Got pregnant	0.0	0	0.6	1

a Percentages do not add to 100 because women could identify more than one reason for discontinuation.

b Challenges with self-injection: inability to do the injection/lack of competence, fear of making a mistake/needles.

c Access challenges: difficulty reaching the clinic, method stockout, or losing the DMPA-SC unit or booklet.

### 3.6 Experience of pregnancy, serious adverse events, side effects and injection-site reactions

There was one suspected pregnancy in the provider-injected group and no suspected pregnancies in the self-injected group. No serious adverse events were reported in either group. Significantly fewer women reported experiencing side effects in the self-injected group at each follow-up interview (Table 5). After the first injection, significantly more women who self-injected experienced an injection-site reaction (e.g., pain, swelling, redness, bump, dimple, or blister), but no differences in injection-site reactions emerged for subsequent injections.

**Table 5:** Self-reported side effects and injection-site reactions (ISR) by self-injected DMPA-SC and provider-injected DMPA-IM users in Senegal

	After 1st injection			After 2nd injection			After 3rd injection		
	Self-injected DMPA-SC (*n*=649)	Provider-injected DMPA-IM (*n*=642)	p value	Self-injected DMPA-SC (*n*=615)	Provider-injected DMPA-IM (*n*=598)	p value	Self-injected DMPA-SC (*n*=588)	Provider-injected DMPA-IM (*n*=559)	p value
Experienced side effects	195 (30.1)	227 (35.4)	.04	130 (21.1)	155 (25.9)	.05	102 (17.4)	125 (22.4)	.03
Sought treatment	18/195 (9.2)	50/227 (22.0)	.00	17/130 (13.1)	32/155 (20.6)	.09	16/102 (15.7)	28/125 (22.4)	.20
Experienced ISR	89 (13.7)	63 (9.8)	.03	52 (8.5)	55 (9.2)	.65	29 (4.9)	30 (5.4)	.74
Sought ISR treatment	0/89 (0.0)	0/63 (0.0)	-	0/52 (0.0)	0/55 (0.0)	-	0/29 (0.0)	1/30 (3.3)	.32

## 4 Discussion

This study provides the first evidence from the francophone West African context that women who self-inject may continue the injectable longer as compared with women receiving injections from providers. The similarity of findings across the three African contexts — Malawi with 12-month continuation of 73% (self-injectors) and 45% (provider-administered DMPA-SC), Uganda with 81% (self-injectors) and 65%, (provider-administered DMPA-IM) and now Senegal with 80% (self-injectors) and 70% (provider-administered DMPA-IM) — is encouraging given the different settings and DMPA products injected [11], [13]. Self-injecting women in Senegal not only continued the injectable longer but demonstrated high injection competency, despite low literacy levels relative to Uganda — about half of women of reproductive age have never been to school in Senegal [2] compared with 10% in Uganda [15]. Collectively, these studies from Senegal, Uganda, Malawi and the United States (the latter two randomized control trials) suggest that offering self-injection as a delivery modality helps women in diverse contexts to continue injectable contraception longer [11], [12], [13].

To a greater extent in Senegal than in Uganda, both self-injectors and women using provider-administered DMPA-IM faced challenges reinjecting on time. Reinjecting late and/or forgetting to reinject (or return for reinjection) accounted for nearly half of the discontinuation cases. The low level of knowledge of reinjection dates, particularly pronounced among women using DMPA-IM (who have significantly lower education, as shown in Table 1), suggests a need to assess provider counseling practices with respect to reinjection timing.

Among self-injectors, difficulty reading the calendar suggests that implementing an automated text or voice reminder system could be beneficial. These approaches have yet to be tested in self-injection programs in low-resource settings. A 2013 systematic review of reminder systems to improve contraceptive adherence — all from the United States — found that just one of three studies demonstrated any benefit (with the successful study showing improved oral contraceptive continuation at 6 months with daily text message reminders) [16]. A second review found just two studies with modest effects: one from Cambodia showing higher self-reported contraceptive use at 4 months (but not at 12 months) among those receiving voice messages and another from the United States showing fewer days between scheduled and actual appointments for injectable users receiving reminder messages for the first, but not subsequent, appointments [17]. The review authors highlight many challenges, including maintaining confidentiality among women using shared phones, poor network coverage, switching phone numbers, cost and communicating accurate information. The latter may be particularly difficult in the context of self-injection since the date for reinjection depends on the date of the previous injection, which is known only to the self-injector. Moreover, in low-resource settings, the women who are most in need of a reminder system might be the least likely to own a cell phone.

Challenges notwithstanding, stakeholders increasingly see the potential for self-injection to reduce unmet need by improving injectable continuation; ministries of health from a dozen countries are developing plans to introduce self-injection as a delivery strategy. As these programs roll out, they create opportunities to explore and test options to increase on-time reinjection adherence, develop cost-efficient training programs, implement effective monitoring systems and design follow-up approaches that provide support and referral to women, consistent with the recent WHO guidelines for self-injection programs [18].

### 4.1 Limitations of the study

This was a nonrandomized study; since participants self-selected into groups, there may be unobserved heterogeneity that affected their continuation. We were unable to systematically track acceptance rates for selection in to groups, so the study cannot speak to the appeal of self-injection relative to provider administration. In addition, our study compared self-injection of DMPA-SC to provider administration of DMPA-IM, which introduced differences in both administration method (self-injection vs. provider injection) and product (DMPA-SC vs. DMPA-IM) between groups. The difference in results could be influenced by perceived or real differences between the DMPA products. That said, two recent research studies in Uganda and Burkina Faso found no differences in continuation rates between DMPA-SC and DMPA-IM when both are administered by providers [19]. Side effects and injection-site reactions were self-reported and not evaluated systematically. Our study was limited in duration and does not address discontinuation after the fourth injection, or how well family planning providers help discontinuing clients to find other contraceptive methods in the event women still wish to postpone or avoid pregnancy.

## Appendix A Supplementary data

Observation checklist used to assess women's competency with self-injection. Image 1
