# Supplementary material for: Continuation of self-injected versus provider-administered contraception in Senegal: a nonrandomized, prospective cohort study
Source: Contraception. 2019 Feb;99(2):137–41. doi: 10.1016/j.contraception.2018.11.001 (PMC6367564; doi:10.1016/j.contraception.2018.11.001)
Supplement: Supplementary file 1 — Observation checklist used to assess women's competency with self-injection. [file mmc1.doc]

## Grille d’observation pour l’auto-injection

| Numéro de la femme | |___|___|___|___|___|___|___| |  | Numéro du site | |___|___| |  |
| --- | --- | --- | --- | --- | --- |
| Code de l’enquêteur | |___|___|___| |  | Date: | |____|____|____| |  |

**Cette grille est utilisée pour évaluer la pratique d'injection de Sayana® Press en utilisant des objectifs des normes de performances objectives. Remettre à la participante le livret d'instructions de Sayana Press à utiliser comme guide au moment de pratiquer l'auto-injection. Les cinq premières observations concernent la pratique sur un préservatif rempli de sel. La sixième observation concerne l'auto-injection surveillée (AI). Pour chaque essai, écrire S (satisfaisant), PS (Pas satisfaisant) ou NE (non effectuée) dans la case correspondant à chaque étape. Les étapes en gras sont essentielles et doivent être correctement faites pour que la femme soit considérée comme compétente.**

| **Etapes** | Observations | | | | | |
| --- | --- | --- | --- | --- | --- | --- |
|  | 1 | 2 | 3 | 4 | 5 | AI |
| 1. Se lave les mains |  |  |  |  |  |  |
| 1. **Choisit un point d’injection approprié et le nettoie si nécessaire** |  |  |  |  |  |  |
| 1. Ouvre la pochette de Sayana Press en déchirant l'encoche. |  |  |  |  |  |  |
| 1. T Tient le dispositif par le porte-aiguille au moment de mélanger le liquide. |  |  |  |  |  |  |
| 1. **Mélange le liquide en secouant vigoureusement le dispositif (environ 30 secondes)** |  |  |  |  |  |  |
| 1. Vérifie que le liquide est bien mélangé et que le dispositif n’est pas abîmé |  |  |  |  |  |  |
| 1. Tiens le dispositif avec l’aiguille dirigée vers le haut durant l’activation |  |  |  |  |  |  |
| 1. Tiens le dispositif par le porte-aiguille pendant l’activation |  |  |  |  |  |  |
| 1. **Enfonce le capuchon sur le porte-aiguille pour activer le dispositif** |  |  |  |  |  |  |
| 1. **Pince la "peau" au point d'injection pour former « un pli ».** |  |  |  |  |  |  |
| 1. Tient le dispositif par le porte aiguille pour piquer la peau |  |  |  |  |  |  |
| 1. Pique l’aiguille dans le pli formé par le pincement de la peau avec le pouce et l’index |  |  |  |  |  |  |
| 1. Enfonce l'aiguille vers le bas. |  |  |  |  |  |  |
| 1. Enfonce complètement l’aiguille de sorte que le porte-aiguille touche la peau |  |  |  |  |  |  |
| 1. Déplace les doigts du porte-aiguille au réservoir tout en continuant à pincer la peau |  |  |  |  |  |  |
| 1. **Presse le réservoir lentement pour injecter le produit - pendant environ 5 à 7 secondes.** |  |  |  |  |  |  |
| 1. Enlève le dispositif du point d’injection tout en continuant de pincer la peau |  |  |  |  |  |  |
| 1. Ne frotte pas le point d’injection. |  |  |  |  |  |  |
| 1. Jette immédiatement le dispositif dans un récipient pour l'élimination d'objets tranchants sans reboucher l'aiguille. |  |  |  |  |  |  |
| **Additionner le nombre total de ‘Ss’ (étape complétée de manière satisfaisante) pour chaque essai et écrire le total dans la colonne à droite.** |  |  |  |  |  |  |

| Selon votre jugement clinique, cette personne a-t-elle démontré suffisamment de compétences pour pouvoir s’auto-injecter en toute indépendance ET a-t-elle effectué correctement les 5 étapes essentielles (en gras) ? OUI NON |
| --- |

Si la réponse est NON, Ne PAS donner à la femme le Sayana Press à emporter à domicile.

- Si la femme n'a pas été capable de s’auto-injecter ou a changé d'avis à propos de l'auto-injection, elle a interrompu l'auto-injection et sa participation à l'étude se termine après l'entretien. faites-lui l'injection et demandez-lui de revenir pour une autre injection dans 3 mois.
- Si elle parvient à s’auto-injecter mais n'est pas compétente, lui demander de revenir à la structure de santé pour une assistance lors de la prochaine autoinjection. Elle peut continuer à participer à la recherche et bénéficier d'une formation supplémentaire quand elle reviendra à la structure de santé dans trois mois.

Dans tous les cas, il faut effectuer l'entretien post-injection.
